# Concerns with computational protein engineering programmes IPRO and OptMAVEn and metabolic pathway engineering programme optStoic

**DOI:** 10.1098/rsob.200173

**Published:** 2021-02-03

**Authors:** Thomas K. Wood

**Affiliations:** Department of Chemical Engineering, Pennsylvania State University, University Park, PA 16802-4400, USA

**Keywords:** protein engineering, modelling, computational pathway engineering

## Abstract

It has become customary in engineering to require a modelling component in research endeavours. In addition, as the code for these models becomes more byzantine in complexity, it is difficult for reviewers and readers to discern their value and understand the underlying code. This opinion piece summarizes the negative experience of the author with the IPRO and OptMAVEn computational protein engineering models as well as problems with the optStoic metabolic pathway model. In our hands, these models often fail to predict reliable ways to engineer proteins and metabolic pathways.

## 1. Review

In the following, I describe our experience with three computational programmes that were designed to improve proteins and metabolic pathways.

### 1.1. Optimal method for antibody variable region engineering (OptMAVEn) for single-chain antibodies

As part of a National Science Foundation grant (CBET 1133040), we investigated the de novo protein design of fully human antibody variable domains for binding a specified antigen using OptMAVEn [1]. In OptMAVEn, possible antigen-binding conformations are generated for a given antigen, then the top scored antigen conformations and antibody models are assembled by combinations of six modular antibody parts and random mutations are introduced to the antibody models for improved antigen-binding affinity.

We focused on trying to improve an existing antibody, 2D10, which is a single-chain antibody (scFv) that recognizes the dodecapeptide DVFYPYPYASGS, a peptide mimic of mannose-containing carbohydrates. As a result of the OptMAVEn predictions [2], we cloned and purified five de novo designed scFvs and verified their correct folding. Of these five, two predicted ScFvs had no binding to the dodecapeptide, and the other three ScFvs had less superior binding by a factor of 2.3–6.2 fold [2]. Hence, in our experience, OptMAVEn was unable to create ScFvs that have superior binding or even equivalent binding.

### 1.2. Iterative protein redesign and optimization procedure (IPRO) for improved dioxygenases

As part of a National Science Foundation grant (BES-0114126), we successfully optimized naphthalene dioxygenase (NDO) of *Ralstonia* sp. strain U2 for oxidizing the benzene ring and the methyl-group of substituted toluene compounds using random protein engineering [3]. We had hoped to use computational methods to help identify additional substitutions that would be beneficial for increasing the activity of the dioxygenase for nitroaromatic pollutants. With this goal, IPRO [4] was performed on the large subunit NagAc of NDO with the aim of optimizing the docking of the substrate 2,3-dinitrotoluene to favour the formation of the intermediate 4-methyl-3-nitrocatechol. IPRO is designed to use energy-based scoring to identify beneficial substitutions in native protein sequences.

Unfortunately, after its construction via site-directed mutagenesis, the IPRO-predicted NagAc variant, L225R/L251I/V258N/F350I, when analysed for 2,3-dinitrotoluene activity by HPLC and a nitrite assay, was completely inactive toward the substrate 2,3-dinitrotoluene. SDS–PAGE analysis showed that this IPRO-predicted variant NagAc L225R/L251I/V258N/F350I was produced at normal levels. Hence, in our experience, IPRO was unable to predict amino acid substitutions that increase protein activity.

### 1.3. IPRO for improved anaerobic oxidation of methane

As part of an Advanced Research Projects Agency–Energy grant, our group reversed methanogenesis for the first time by engineering an archaeal strain so that it could grow on methane as a pure culture [5]. This required cloning methyl-coenzyme M reductase (Mcr) from an unculturable organism (which was part of an anaerobic methanotrophic archaeal population in a Black Sea mat) into *Methanosarcina acetivorans* and using 10 mM FeCl3 as an electron acceptor [5]. This led to the engineered archaeal strain that could produce acetate [5], lactate [6] and electricity [7].

As part of this project, we tested the IPRO predictions for engineering Mcr, the enzyme for capturing methane as part of reversed methanogenesis, by assaying for improved enzyme kinetics (i.e. greater consumption of methane). Critically, we tested the top two model predictions for engineering McrA to improve F430 cofactor binding. After sequencing to confirm the plasmid constructs were correct, we tested McrA V419 K and found the predicted substitution not only did not improve methane capture, it abolished methane capture (i.e. it inactivated the enzyme). We also tested the predicted substitutions McrA M78R/H157D/V419 K and found these predictions also abolished methane capture. Hence, in our hands, IPRO is unable to improve Mcr.

### 1.4. Optimum overall stoichiometry (optStoic) de novo metabolic pathway modelling

As noted in the previous section, our laboratory discovered how to reverse methanogenesis by using the external electron acceptor ferric iron, to generate acetate in the autumn of 2014; these results were subsequently published in 2016 by our group [5]. To our surprise, our laboratory results on using ferric iron to reverse methanogenesis with cloned Mcr in an archaeal strain were published without our approval and without acknowledgement as a means to give an example of the power of the optStoic metabolic pathway modelling routine [8]. optStoic is designed to identify the optimum overall stoichiometry that maximizes carbon, energy or price efficiency based on thermodynamic constraints. Our laboratory research results were known to the group that published them since this group was part of the same ARPA-E research grant and received monthly reports. Strikingly, they used our laboratory results as one of the examples to demonstrate the robustness of their ‘model’ prior to our own publication of these results.

The common features between our proven laboratory discovery and their ‘modelling predictions' include: (i) anaerobic capture of methane for growth by a pure culture (which had not been demonstrated previously); (ii) methane conversion to the same end product, acetate (iii) metabolism based on the same electron acceptor, ferric iron; (iv) metabolism based on the same enzyme, Mcr; and (v) metabolism based on the identical archaeal host, *M. acetivorans.* Hence, the model ‘predictions’ (e.g. the importance of ferric iron, generation of acetate) were published using our known, albeit unacknowledged, experimental results.

## 2. Perspectives

The main conclusion from our use of the IPRO and OptMAVEn protein engineering models is that they are often incapable of predicting substitutions that improve protein function. Hence, evaluation of these kinds of models should be predicated on a positive control being performed in which the model predicts *a priori* some known beneficial substitution that is experimentally verified. It should be noted that other *in silico* protein engineering approaches exist, such as computer-aided directed evolution of enzymes [9], which successfully screened the effect of 128 substitutions in triosephosphate isomerase from *Saccaromyces cerevisiae.* Moreover, a Rosetta enzyme design approach for sampling the sequence and conformational space of partial active site randomization led to the synthesis of four chiral β-amino acids via an aspartase from *Bacillus* sp. YM55-1, without laboratory evolution [10].

In addition, it is worth noting that without a doubt, experimentalists often get it wrong, too, without the aid of protein modellers. For example, it has been predicted in a prestigious journal that the archaeal strain *M. acetivorans* can grow on methane faster than *Escherichia coli* can grow on glucose [11], which is highly unlikely.

Furthermore, with 19 possible substitutions for every amino acid and an average protein size of 333 aa, there are 19^333^ possible substitutions, so the protein space for models (and experimentalists) have to cover is enormous. However, of the two approaches for protein engineering, experimental random mutagenesis versus computational protein engineering, a Nobel Prize has been given only for the experimental approach [12]. Moreover, over two decades, we have had success in using random protein engineering to create better catalysts and regulators by using DNA shuffling with monooxygenases [13–17], dioxygenases [3], epoxide hydrolases [18], toxin/antitoxin systems [19] and biofilm regulators [20–23]. However, we have never had success in using computational protein engineering for improving either enzyme activity or protein binding. The crux is that these computational models should be vetted more thoroughly before their predictions are accepted, and experimentalists should use these programmes with discretion.

## Data accessibility

This article has no additional data.
